# Surface-Bound and Volatile Mo Oxides Produced During Oxidation of Single MoS_2_ Crystals in Air and High Relative Humidity

**DOI:** 10.3390/ma13143067

**Published:** 2020-07-09

**Authors:** Robert Szoszkiewicz, Maciej Rogala, Paweł Dąbrowski

**Affiliations:** 1 Faculty of Chemistry, Biological and Chemical Research Centre, University of Warsaw, Żwirki i Wigury 101, 02-089 Warsaw, Poland; 2 Department of Solid State Physics, Faculty of Physics and Applied Informatics, University of Lodz, Pomorska 149/153, 90-236 Lodz, Poland; rogala@uni.lodz.pl (M.R.); pawel.dabrowski@uni.lodz.pl (P.D.)

**Keywords:** MoS_2_, MoO_3_, surface science, nanoscale ripples, atomic force microscopy, XPS

## Abstract

We report on the MoO_3_ oxides and their derivatives on microscopic 2H MoS_2_ flakes oxidized in air and high relative humidity at a moderate temperature range below 410 °C. We combine XPS and AFM measurements such as topography, friction, creation of nanoscale ripples and scratches on the MoS_2_ flakes deposited on Si substrates. We detect MoO_3_ oxides mostly by measuring selected nanomechanical properties of the MoO_3_ layer, such as its compressive mechanical stress at the plastic yield. We discuss basal surface coverage of the single MoS_2_ flakes by the MoO_3_ oxides. We discuss conditions for appearance of all possible MoO_3_ oxide derivatives, such as molybdenum(VI) hydroxyoxides and MoO_3_ hydrates. Our findings agree with an expected mechanistic switch in thermal oxidation in water vapors vs. air.

## 1. Introduction

Naturally occurring MoS_2_ crystals, molybdenites, have been widely used as solid lubricants [1]. The most common 2H molybdenite has three other polytypes: 3R, 1T and 1T’, which differ in structure and electronic properties [2,3]. 2H and 3R molybdenites can be easily peeled off mechanically to yield atomically flat MoS_2_ crystals with thickness down to one monolayer [2]. Easiness of mechanical exfoliation and semiconducting properties of 2H MoS_2_ crystals have opened up a possibility to use them in a new generation of thin transistors [4]. Today, thin 2H MoS_2_ crystals contribute tremendously to vigorous growth of flexible nanoelectronics, particularly in sensing, optoelectronics and energy harvesting [2,5,6,7]. In order to exploit all of these applications, the surface reactivity of single 2H MoS_2_ microscopic flakes needs to be understood. The simplest and most widely applicable surface reactions pertain to their oxidation in ambient conditions and in the presence of water.

It has been confirmed both experimentally and theoretically that bulk MoS_2_ oxidation is not readily observed at ambient conditions due to high energy barriers for such reactions [8,9,10,11,12,13,14,15]. Nevertheless, all kinds of thin and thick microscopic MoS_2_ crystals are etched by oxygen within the time scales of minutes when heated to temperatures of at least 320 °C. Such etching progresses according to a following stoichiometry [8,9,10,11,12]:

2MoS_2_ + 7O_2_ → 2MoO_3_ + 4SO_2_(g) (1)

Equation (1) states that out of all the possible Mo oxides, only the MoO_3_ is produced. Exclusive presence of MoO_3_ on the oxidized MoS_2_ samples has been observed in many oxidative processes carried out in oxygen or in air. Transparent MoO_3_ crystals change into yellow and grayish-blue, only when oxygen defects yielding Mo^5+^ and Mo^4+^ species, respectively, are purposefully introduced, e.g., via hydrogen adsorption [16]. XPS and XRD experiments on thin Mo films have shown that all defective MoO_3_ species with Mo^4+^ and Mo^5+^ became fully oxidized to MoO_3_ already at more than 5% of oxygen in reactive gases [17]. The majority of the published studies, mostly using the XPS measurements, detect exclusively Mo^6+^ oxides—i.e., MoO_3_, onto thermally oxidized MoS_2_ samples [12,18,19]. Furthermore, by performing thermodynamic calculations and using enthalpies of bulk reactions, Walter et al. have calculated that for a closed system consisting of the MoS_2_ crystals in air, Equation (1) is indeed an exclusive reaction pathway, but only till system temperature of 470 °C [12]. Above 470 °C, volatile (MoO_3_)_3_ and (MoO_3_)_4_ species started to appear in their simulations due to sublimation of the MoO_3_(s). At 525 °C none of the MoO_3_ was predicted to stay on the oxidized MoS_2_ samples.

Somewhat different MoS_2_ oxidation outcomes, however, are expected in the presence of water or humid air. Ross and Sussman experimented with pulverized bulk MoS_2_ crystals and showed that water vapors helped in producing substantially more MoO_3_ than expected from Equation (1). They verified that an additional MoO_3_ was produced in the course of a following reaction [20]:

2MoS_2_ + 9O_2_ + 4H_2_O → 2MoO_3_ + 4H_2_SO_4_ (2)

Furthermore, volatile molybdenum(VI) hydroxyoxides, MoO_2_(OH)_2_(g), have been predicted to appear above 300 °C in the calculations of Walter et al. (There, partial pressure of water vapors was set to the saturared water vapor pressure at room temperature, or to 100% relative humidity at 25 °C (298K)) [12]. It is not clear whether MoO_2_(OH)_2_ originated directly from water-mediated oxidation of the MoS_2_ crystals or rather from water reacting with the MoO_3_ adsorbed on the MoS_2_ crystals, which seemed more likely. Noteworthily, bulk MoO_3_ has been shown by inorganic chemistry books to dissolve slightly in water. Finally, yet other Mo species, the MoO_3_ hydrates such as MoO_3_·H_2_O, have been recently suggested to appear on the MoS_2_ crystals after their immersion in water for many hours [21]. 

Not only is the chemistry of the MoO_3_ complicated, but its appearance and surface distribution on microscopic 2H MoS_2_ crystals are elusive too. In the case of very thin MoS_2_ flakes some loose islands of the MoO_3_ oxides have been presumably observed directly in the AFM topographs overlaid with AFM-based magnetic force imaging [9]. Therein, MoO_3_ was reported to be a non-magnetic material. In one of our earlier AFM and micro-Raman studies we pointed out that during initial 10–15 min of heating at temperatures ranging from 320 to 390 °C, in air, a predominantly observed outcome on thick microscopic MoS_2_ flakes was triangular etch pits within the basal MoS_2_ surface [11]. Similar triangular etch pits were observed several years earlier on very thin MoS_2_ samples heated either in oxygen or in oxygen/argon flow or in air [8,9,10]. In all of those studies a typical depth of triangular etch pits was exactly one MoS_2_ monolayer; i.e., 0.7 nm. Beyond triangular etch pits, very few morphological changes of the MoS_2_ flakes have been noticed [11]. Since MoO_3_ crystals are transparent, it is not clear whether MoO_3_ stayed with those samples at all and/or covered the pits and a surrounding area uniformly. Particularly, almost the same thickness is expected for the MoO_3_ and MoS_2_ single layers [22,23,24]. 

It is not only difficult to detect MoO_3_ via its physical properties, but according to our knowledge, none of the published results provided a direct chemical proof of the local MoO_3_ existence on a single microscopic 2H MoS_2_ flake. Typical XPS measurements are not so local and encompass at least several MoS_2_ flakes. Raman measurements do not help either. The majority of the published Raman studies show either miniscule shifts of the Raman modes fingerprinting the MoS_2_ crystals, i.e., the A_1g_ and E^1^_2g_ modes [10,11], or an exclusive presence of the MoO_3_ crystals on fully oxidized single 2H MoS_2_ flakes [8]. This is likely due to a very small thickness of the oxide layer. Overall, the MoO_3_ layers produced during gentle oxidation of single 2H MoS_2_ crystals are transparent, thin and difficult to be differentiated both physically and chemically from an underlying MoS_2_ substrate. In addition, such MoO_3_ layers might contain MoO_3_ derivatives, such as molybdenum(VI) hydroxyoxides and/or MoO_3_ hydrates. Clearly, proper identification and surface distribution of the MoO_3_ species and their derivatives on single 2H MoS_2_ flakes deserve substantially more attention. 

In this study we detected and differentiated the MoO_3_ oxides and their derivatives from an underlying MoS_2_ crystal on single and microscopic 2H MoS_2_ flakes. We used a series of the MoS_2_ samples, which were oxidized gently, each one at a different temperature between 205 to 410 °C, in dry or humid air. First, using XPS measurements we show how the content of the MoO_3_ species on the MoS_2_ samples changes with oxidization temperature. Next, by carefully manipulating AFM tips we created nanoscale ripples within the MoO_3_ layer and scratched them out from single MoS_2_ flakes. We related the thickness of the scratched out MoO_3_ layer to its structure and used a model for formation of nanoscale ripples to obtain its compressive breaking strength. Next, we proved that oxidation on the edges of the MoS_2_ crystals provides most of the needed MoO_3_ to cover the MoS_2_ flakes. Finally, we study sublimation of the MoO_3_ oxide layer and its formation in high relative humidity conditions. 

## 2. Materials and Methods

Preparation of MoS_2_ flakes: Laboratory grade 2H molybdenite crystals were bought from SPI Supplies, West Chester, PA, USA, catalogue number #429MM-AB. The MoS_2_ samples have been mechanically exfoliated using a standard double-sided scotch tape and transferred on fine polished and basically undoped <111> Si crystals with resistivity more than 10,000 Ω cm, bought from ITME, Warsaw, Poland. Prior to a sample transfer, the Si substrates were ultrasonically cleaned with acetone and isopropanol and dried with pure N_2_. Due to subsequent heating studies at temperatures of more than 300 °C, no annealing nor other cleaning methods have been used to remove any remaining traces of the scotch tape from the Si substrates. For sample heating in air we used a standard hot plate covered with a quartz Petri dish to insure a controlled atmosphere and proper distribution of temperatures within a heating zone. After calibrating the hotplate with a standard thermocouple and a Pt thermometer, we established a temperature error of ± 2K on the sample surface. For experiments in high relative humidity we used a custom-built chemical reactor.

X-ray photoelectron spectroscopy (XPS): XPS was conducted in the multi chamber ultrahigh vacuum system (manufactured by Omicron Nanotechnology, Taunusstein, Germany) equipped with a hemispherical energy analyzer Phoibos 150 (manufactured by SPECS, Berlin, Germany) with a 2D-CCD detector. DAR 400 X-ray lamp with a non-monochromatic radiation of 1253.64 eV (Mg Kα) was used. The spectra for analysis of chemical shifts in Mo 3d core lines were collected with pass energy of 30 eV in “Fixed Analyzer Transmission” mode. For the peak fitting procedure, the CasaXPS (v.2.3, Casa Software Ltd., Teignmouth, UK) software was used. To ensure repeatability of the analysis of all samples, the same set of components with fixed energy separation was used for each of them. The binding energies of the measured spectra were calibrated to the C 1s core line position at 285 eV. For all of the investigated samples, an observed charging effect did not exceed 2 eV and no hardware charge compensation was needed.

Atomic force microscopy (AFM): Non-contact/tapping AFM imaging was conducted at amplitudes of several tens of nanometers with AC-160TSA-R3 cantilevers from Olympus, Tokyo, Japan using Cypher-S AFM manufactured by Asylum Research, Goleta, CA, USA. Contact mode AFM imaging was conducted with MLCT-E and/or MLCT-F cantilevers from Bruker, Santa Barbara, CA, USA and using Dimension Icon AFM manufactured by Bruker. We collected raw topography and lateral force microscopy (LFM) images of the investigated MoS_2_ flakes. The images were collected with at least 256 points per line and treated with Gwyddion (v.2.51, Czech Metrology Institute, Brno, Czech Republic) software [25]. On topography images we removed glitches and used standard line-by-line first or second-order flattening and three-point plane levelling methods. No other image treatment and/or conditioning was performed. 

Lateral spring constant calculations for AFM cantilevers used for measurements of the compressive breaking strength. The value of the lateral stiffness *k_lat_* for a triangular MLCT-E cantilever used to indent and scratch the oxidized MoS_2_ crystals was calculated using the Neumeister and Ducker model [26]. Unless otherwise measured from optical microscopy images, we used manufacturer’s specifications for all the relevant variables; i.e., SiN_x_ Young modulus of 304 GPa, Poisson ratio of 0.24, thickness of a cantilever of 0.60 ± 0.05 μm, triangle opening angle of 28 ± 2° (measured), length of cantilever’s arms from the base towards the point they meet of 105 ± 5 μm (measured), distance from the tip to the edge of a cantilever 4 ± 1 μm, width of cantilever arms of 18 ± 1 μm (measured) and tip’s height of 7 ± 1 μm. Those values yielded *k_lat_* of 54 N/m. Considering the errors of each parameters we obtained a minimum allowable *k_lat_* of 25 N/m and a maximum of 116 N/m. These values of *k_lat_* have been calculated using such a combination of parameters (with their errors) to obtain the lowest and the largest values of *k_lat_*, respectively. Thus, we reported an average value within the errors; i.e., *k_lat_* = 71 ± 45 N/m. Using the Neumeister and Ducker approach, we also obtained a normal spring constant of 0.097 N/m, which is typical for the MLCT-E levers.

## 3. Results and Discussion

**Figure 1:**
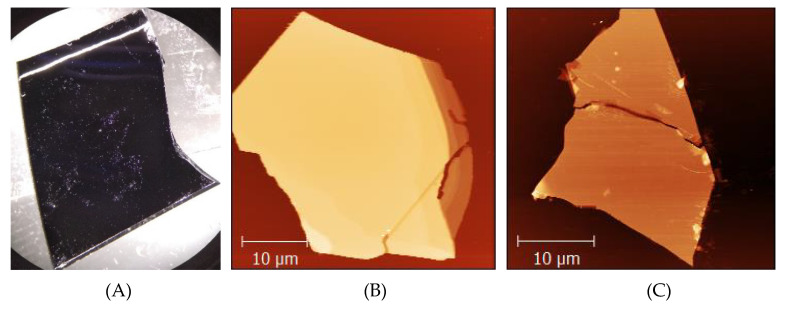
An exemplary sample containing agglomerates of mostly thick mechanically exfoliated 2H MoS_2_ crystals on a silicon substrate. (**A**) An optical microscopy image of a typical sample being ca. 2 cm long and ca. 1.5 cm wide. Visible whitish spots are agglomerates of single MoS_2_ flakes. Two of such single MoS_2_ flakes are presented in (**B**,**C**). Image (**B**) is an AFM topograph of a flake with a mean height of a central zone (excluding several lone MoS_2_ pieces) of ca. 65 nm and a Z-scale of the image of 130 nm. (**C**) An AFM topograph of another flake with a mean height of a central zone of ca. 25 nm and a Z-scale of the image of ca. 65 nm. For images (**B**,**C**) brighter colors represent portions of the image, which are higher (Z-scale).

To investigate the presence of Mo oxides on the MoS_2_ crystals, we prepared single 2H MoS_2_ crystals on silicon/silica substrates, as explained in the Materials and Methods. We have studied flakes thicker than 10 nm, i.e., of more than 15 MoS_2_ monolayers due to known dependencies of several physico-chemical properties of the MoS_2_ flakes on their thickness, particularly for the flakes thinner than 10 monolayers [27]. First, the MoS_2_ flakes were localized using light microscopy and AFM; see Figure 1. Next, we selected five samples with MoS_2_ flakes for the XPS studies of their thermal oxidation. One of the samples served as our reference sample. Four other samples were separately heated on a hot plate for 10–15 min each: first at 320, second at 350, third at 370 and fourth at 410 °C.

**Figure 2:**
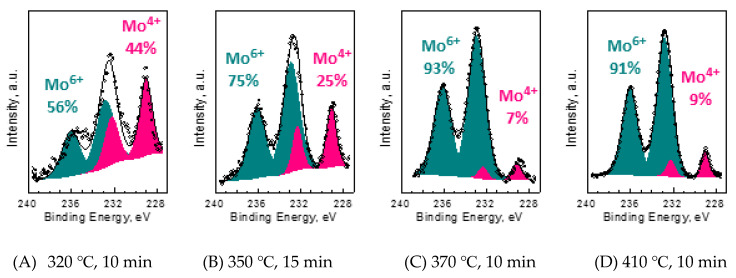
Our XPS results. (**A**–**D**) The XPS Mo 3d core line spectra of MoS_2_ flakes heated in different temperatures presented together with the charts showing the concentrations of Mo^4+^ and Mo^6+^ ions within the surface layer of the flakes. The contributions coming from different oxidation states were fitted to the spectra and are presented in the charts below.

Figure 2 shows the results of our XPS investigations on the samples prepared and oxidized in air. The Mo 3d core line XPS spectra were used in order to probe an oxidation process. Each such XPS spectrum contains four maxima, which have been related to the presence of two different oxidation states of the Mo ions. The applied peak fitting procedure yielded the binding energies of each maxima at 229.0 eV, 232.2 eV, 232.8 eV and 236.0 eV, respectively. The first two maxima were related to the spin-orbit doublet (Mo 3d 5/2 and 3/2) of the Mo^4+^ oxidation state, which is characteristic for the MoS_2_ sample. The second two maxima were related to the presence of the Mo^6+^ oxidation state doublet, which within our reaction conditions has been predicted to be characteristic of the MoO_3_. During peak fitting procedures, the Gaussian–Lorentzian peak shapes were used and the relative peak areas were fixed to a 2:3 ratios for each set of the respective 3/2 and 5/2 pairs. This procedure and the resulting binding energies are consistent with previous works [12,18,19]. 

The oxidation of a single MoS_2_ flake has been suggested to start already above 200 °C, as inferred from electronic density shifts within the MoS_2_ monolayers via micro-Raman measurements [8]. However, earlier reports have shown through visualization of the obtained triangular etch pits that meaningful MoS_2_ oxidation occurs in air only above 320 °C [8,9,10,11,28]. Such conclusions agree our XPS analysis in Figure 2. The presented results clearly show that during heating to a certain temperature the concentration of the Mo^4+^ ions decrease, which is visible when comparing the spectra after heating at 320, 350 and 370 °C; see Figure 2A–C. However, somewhere above 370 °C this trend is stopped or even reversed, as is visible in the XPS spectra obtained after heating the sample in 410 °C; see Figure 2D. The XPS results, however, are averaged over an investigated area, which is typically between several tens to a hundred of µm^2^. Therefore, we decided to investigate the distribution of the Mo oxides on the surface of MoS_2_ flakes using local AFM measurements. 

After many trials and using high resolution, contact non-contact/tapping AFM imaging, we could not detect any obvious MoO_3_ islands on and/or in the vicinity of the triangular etch pits on the XPS studied samples till heating them at 370 °C. We did not quit searching for the surface-bound MoO_3_ layers on the prepared batch of samples; XPS results in Figure 2 clearly show the prevailing presence of oxides on the samples heated between 350 and 410 °C. Furthermore, the XPS results did not change upon sample annealing at 210 °C for 30 min, confirming that the Mo oxide stayed there even after annealing. This is why we decided to locally scratch several samples heated between 350 and 370 °C. Indeed, after several passages of a scanning AFM tip, we managed, at least in several cases, to break through a complete layer of the likely MoO_3_ oxide after applying normal forces of up to several nN. While continuing the scratching process, we managed to remove any remaining pieces of the scratched layer and to expose a fresh basal plane of the Mo disulfide. 

**Figure 3:**
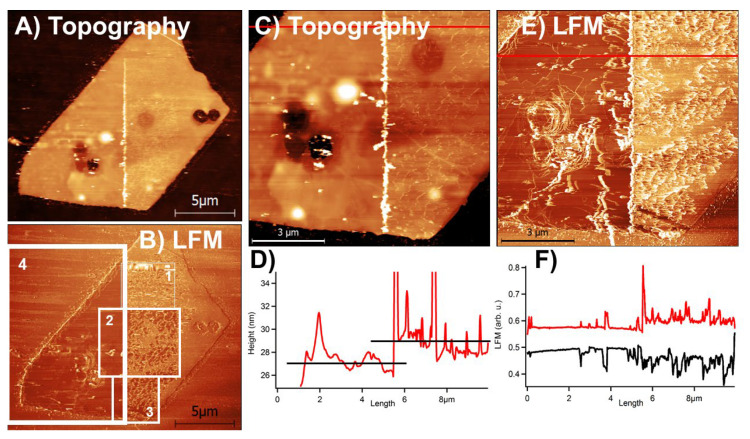
Removing the surface-bound MoO_3_ oxide layer on a single MoS_2_ flake with an AFM tip. The images show AFM recorded topography for (**A**,**C**) and uncalibrated friction (LFM signal) for (**B**,**E**). (**B**) shows that the flake was continuously scratched in zones 1, 2, 3 and 4 with a progressively increasing number of rescans between the zones. In the zone “4” the flake was rescanned more than 10 times, which produced complete removal of the oxide layer. (**C**) shows a close-up on the topography data from (**A**). (**D**) presents a topography cross-section line, marked in red in (**C**), to show that a removed oxide layer was 2 ± 1 nm thick. (**E**) shows an LFM signal corresponding to (**C**). **(F)** shows a friction loop along a cross-sectional line presented in red in (**E**). A friction loop is an LFM signal recorded in trace (R− > L) and retrace (L− > R) scanning. It corresponds to twice of the uncalibrated friction. For (**A**,**C**) brighter colors represent higher portions of the image (Z-scale), while for (**B**,**E**) brighter colors represent higher values of the lateral force microscopy (LFM) signal. Z-scale in (**A**) was 60 nm or (**C**) 43 nm.

Figure 3 presents the results of removing a surface-bound MoO_3_ oxide layer with help of an AFM tip. It shows the AFM recorded surface topography and corresponding lateral force microscopy (LFM) signals to visualize changes provoked on the sample during scratching. The investigated MoS_2_ flake was ca. 30 nm thick. It was oxidized in five cycles of 10-minute heating at 350 °C and subsequent cooling to room temperature. The oxidized flake was continuously scratched in the zones denoted respectively by numbers “1,” “2,” “3” and “4” in Figure 3b. In the zone “1” the MoS_2_ flake was rastered several times only, but it was enough to break continuity of the top oxide layer and produce lasting and visible indents in this layer. In the zones “2” and “3” the flake was scratched several more times than in “1,” so that visible surface ripples appeared within the oxidized MoS_2_ surface. In the zone “4” a portion of the MoS2 flake was rescanned more than 10 times, resulting in a complete removal of an oxide layer. 

AFM topographs cannot provide direct information about the local chemistry of the investigated samples. Nevertheless, with their help we can discuss the thickness of the scratched out MoO_3_ layer. To start with, after averaging several cross-sectional lines in local surface topography in Figure 3c, we obtained that a removed oxide layer was 2 ± 1 nm in height; see Figure 3d. 

Next, we explain how an obtained thickness of the oxide layer corresponds to its structure. The most thermodynamically stable MoO_3_ polymorph existing in nature is the α-MoO_3_ called molybdite. Refined molybdite crystal structure shows a true layered arrangement of the MoO_6_ octahedra with an orthorhombic unit cell belonging to a *Pbnm* space group [24]. Any given MoO_3_ layer is a double layer composed of MoO_6_ octahedra at two height levels. Within each level the MoO_6_ octahedra are connected by shared lateral ends only. Connections between a ground level and an upper level are realized by sharing one edge between any two octahedra from ground and upper levels, respectively. Such an arrangement yields an overall height of a single α-MoO_3_ layer, *h*, it being 3/2 of the height of a single MoO_6_ octahedron plus any necessary spacing between the layers. Consequently, we estimate *h* to be ca. 0.8 nm [24]. On the other hand, we can also estimate the value of *h* differently. To provide for dense spatial packing within the α-MoO_3_ structure, a second (upper) α-MoO_3_ layer is laterally shifted with respect to a first (lower) layer, but a third MoO_3_ layer is positioned like a first layer. Such an arrangement yields the height of a respective α-MoO_3_ unit cell stretching from first to third layer to be 1.4 nm. The thickness of an α-MoO_3_ monolayer atop another α-MoO_3_ monolayer is half of this value; i.e., 0.7 nm. Again, a slightly larger value is expected for the height of the α-MoO_3_ monolayer on the 2H MoS_2_ crystal due to non-matching lateral dimensions between α-MoO_3_ and 2H MoS_2_. Thus, the expected thickness of a single MoO_3_ layer atop the MoS_2_ basal surface is between 0.7 and 0.8 nm, which makes it extremely close to the thickness of a single MoS_2_ monolayer of ca. 0.7 nm. Consequently, the detected thickness of the MoO_3_ layer corresponds to something between one to four MoO_3_ monolayers.

Another important difference between MoS_2_ crystals and detected MoO_3_ oxide layers observed in Figure 3e,f is the substantially larger, ca. 1.5 times larger, friction on the surface containing rippled oxides comparing to a cleaned MoS_2_ surface. A substantial friction increase was expected on the oxidized vs. pristine MoS_2_ surface. MoS_2_ surfaces are known for low friction and their moderately hydrophobic behavior acquired from the pristine MoS_2_ surface after its quick passivation by hydrocarbons [29]. On the contrary, Mo oxides and their derivatives are strongly hydrophilic, which facilitates formation of water capillary bridges between these surfaces and a scanning AFM tip in ambient conditions. Such capillary bridges are known to account for substantial friction increase in air [30,31,32].

The results are presented in Figure 3, and more of such results together with accompanying high resolution XPS spectra, presented in the Supplementary Materials (Figures S1 and S2), confirm that the Mo oxide layer can be indented and scratched away from MoS_2_ basal planes of single MoS_2_ crystals. Let *σ_MoO3_* denote the compressive mechanical stress at the plastic yield of that layer. It is also referred to as the compressive breaking strength. The value of *σ_MoO3_* can be estimated from our experimental results presented in Figure 3b using the Dugdale model of pressure-induced formation of cracks in solid surfaces [33], which was adopted for conditions of indenting a material with a scanning AFM tip [34]. In brief, the model assumes that a scanning AFM tip performs a stick-and-slip motion between the cracks, which are generated just underneath the very surface of a material and become visible within the indents within the material created by an AFM tip. Any given indent grows when a tip of an AFM cantilever “sticks” to it by exerting a normal load. At the same time the tip shifts laterally in the process of surface scanning. Thus, the tip will “slip” by the lateral distance, *Δ*, to a new indentation point once mechanical energy stored in the cantilever, due to its increasing lateral bending, exceeds the tip-material surface energy over the contact area. The value of *Δ* is later measured as the distance between consecutive indents. The lateral length of a given indent within a material is denoted as *δ_t_*. The local breaking strength of a material, *σ*, is calculated from a following equation:

*σ* = (0.5 · *Δ*^2^ · *k_lat_*)/(1.2 · 2 · π · *R* · (*D* + *H*) · *δ_t_*) = 0.0663 · *Δ*^2^ · *k_lat_* / (*R* · (*D* + *H*)· *δ_t_*) (3)

In Equation (3), *D* is the depth of a crack; *H* is the height of a material pile removed during indentation within a crack; *R* is the tip curvature radius of an AFM cantilever and *k_lat_* is its lateral (elastic) stiffness. 

After careful examination of the zones “1” and “2” in Figure 3b we have observed that the values of *Δ* and *δ_t_* are similar and typically equal several hundreds of nanometers. Following this observation we simplify Equation (3) to yield: *σ_MoO3_* = 0.0663(*Δ* · *k_lat_*) / (*R* · (*D* + *H*)). The values of (*D* + *H*) are limited in our case by a small thickness of an oxide layer. We have estimated (*D* + *H*) *=* 5 ± 2 nm; see Figure 3b. For the used here MLCT-E cantilever, *R* was estimated as 50 ± 10 nm and *k_lat_* = 71 ± 45 N/m was obtained, as explained in the Materials and Methods. Consequently, we obtained values of *σ_MoO3_* between 0.3 and 7.8 GPa. An upper limit of ca. 8 GPa is likely overestimated, since our values of (*D* + *H*) are smaller than in the case of a thick MoO_3_ layer. 

The value of *σ_MoO3_* can be explained quite well using some previously published data and our experimental details. We did not find the value of *σ_MoO3_* in the literature; however, the MoO_6_ octahedra within each single MoO_3_ layer are slightly deformable under pressure along one of the lateral directions [24]. On the contrary, the MoS_2_ surface exhibits nice honeycomb lattices within each of the respective S and Mo-planes, which are not easy to break through [22,23]. Thus, one expects the value of *σ_MoO3_* to be much lower than a respective value of *σ_MoS2_*. In fact, within our experimental conditions, we did not observe any breaking through the underlying MoS_2_ layer under the maximum loading forces applied. Those forces were of up to four times of the loads applied to generate scratches presented in Figure 3. Therefore, up to several-times-lower values of *σ_MoO3_* than *σ_MoS2_* are expected. Several published experimental and theoretical studies reported the values of *σ_MoS2_* between 20 and 30 GPa [35,36]. Consequently, our values of *σ_MoO3_* might indeed relate to the presence of thin α-MoO_3_ layers. In conclusion, despite not being easily noticeable, a thin α-MoO_3_ layer can be differentiated via creating surface ripples and scratching out the oxide layer via repetitive scanning on the MoS_2_ samples with appropriate AFM cantilevers. 

**Figure 4:**
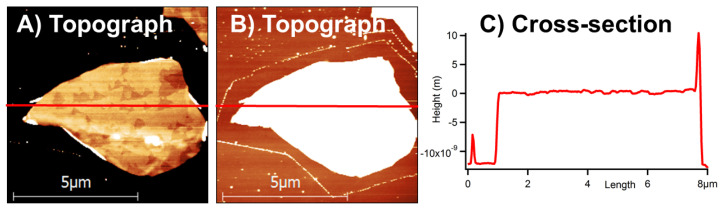
Etching and shrinking of a single MoS_2_ flake. (**A**,**B**) show topography images of the flake, with the Z-scale of several nanometers only in each. (**A**) is centered on a basal flake surface and (**B**) is centered on a surface of the silicon substrate. (**C**) presents a topographical cross-section taken laterally in a middle of the flake, as shown in A and B, in red. The flake is 12 ± 2 nm thick.

Next, we address the origins of the Mo oxide layer, which deposits on the MoS_2_ surfaces. Etched triangular pits would account for only a very small amount of the produced oxides and there must be other sources of it. In Figure 4 one can see an example of the MoS_2_ flake with MoS_2_ monolayers of thickness between 15 and 20 depending on a position along the flake. The flake was imaged at ambient conditions just after 12 min of its thermal oxidation at 390 °C in air. One can clearly notice triangular etch pits within the basal surface of the flake in Figure 4a, and a clean area surrounding the bottom of the flake in Figure 4b. 

The clean area around the MoS_2_ flake observed in Figure 4b has been freshly produced, since it contains no spots. This should be compared with many small spots visible on the Si substrate due to its prolonged handling in air. Thus, such a clean area must have originated from lateral shrinking of the flake during thermal oxidation. At the same time, not too many triangular etch pits were produced on this flake; see Figure 4a. Consequently, the amount of the Mo oxides produced by volumetric oxidation at edges of the flake must was substantially greater than the amount of oxides produced from its basal surface oxidation. Thus, oxidation on edges of the MoS_2_ flakes is a primary source of the MoO_3_, particularly at heating temperatures of 350 °C and above. This result agrees with already published observations made by angle-resolved XPS, but on macroscopic MoS_2_ crystallites [37]. We have observed similar effects on many other MoS_2_ flakes, which were not too thick. On thick MoS_2_ flakes this effect is barely visible due to substantial volume of the MoS_2_ material enclosed within only slight shrinking of the lateral dimensions of such flakes due to heating. 

Consequently, the only way to reconcile our data, i.e., volumetric oxidation, triangular surface etching and formation of an eventual thin Mo oxide layer, is to suggest that freshly-produced MoO_3_ must escape directly to the gas phase, but some portion of it can deposit back on the surface to form islands and even organized layers under appropriate conditions. In this sense, we have validated microscopically that the MoO_3_ produced through Equation (1) is initially in the gas form, at least within our experimental conditions.

Following the hypothesis that originally produced MoO_3_ goes to the gas phase first, we continued to notice that the amount of the MoO_3_ oxides, which are deposited back on the MoS_2_ surfaces, decreases as oxidation temperature rises above 370 °C; see Figure 3e. While more “energetic”—i.e., produced at higher temperatures—MoO_3_ particles in the gas phase have less of a tendency for surface adsorption, such results admit as well a possibility that the MoO_3_ particles, which have adsorbed on the surface, might quickly sublimate at those conditions. Indeed, high quality MoO_3_ crystals grown on gold have been showed to sublimate above 400 °C [38] and monolayers of amorphous MoO_3_ grown on MoS_2_ via O_2_ plasma exposure have sublimated completely at 500 °C [39]. Furthermore, thermodynamic calculations of Walter et al. [12] predicted MoO_3_ sublimation to start above 470 °C. Below, we provide an experimental proof for sublimation of the surface-bound MoO_3_ layers already at 320 °C. 

**Figure 5:**
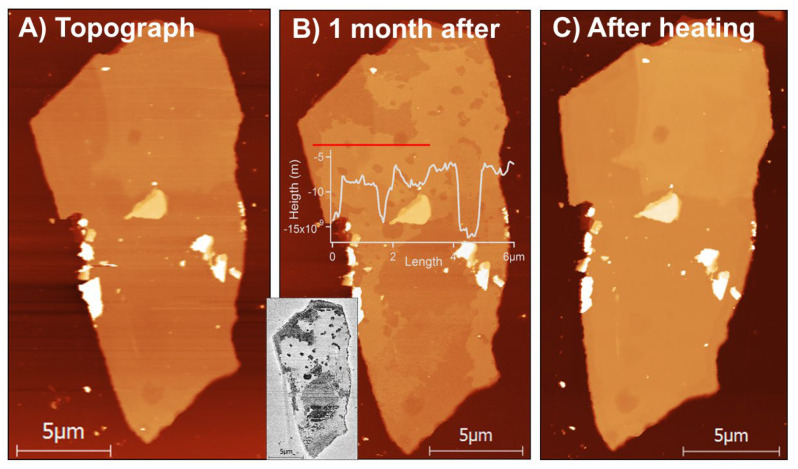
Sublimation of Mo oxide layers. (**A**) shows an initial MoS_2_ flake obtained by AFM imaging in non-contact mode. (**B**) presents additional build-ups, likely MoO_3_ layers, deposited on various portions of the flake after its short oxidation at 370 °C followed by one month stay in humid desiccator. A marked cross-sectional line (in red) shows that a typical thickness of an oxide layer is ca. 5 nm. Inset: corresponding AFM phase imaging, which seems to differentiate roughly oxidized (white) vs. non-oxidized (gray) portions of the flake, at least on a top part of the flake. (**C**) shows that the entire oxide layer sublimated after the sample was heated at 320 °C for only 4 minutes. Z-scale in (**A**) was 132 nm, in (**B**) 129 nm and in (**C**) 90 nm.

Figure 5 presents an MoS_2_ flake heated for several minutes at 370 °C to promote oxidation and then left for about a month inside of a humid desiccator. In Figure 5a one can see an initial MoS_2_ flake. In Figure 5b one observes clearly some additional build-ups, likely MoO_3_ layers, which have deposited on various portions of the flake after its prolonged stay in desiccator. In Figure 5c one can notice that an entire oxide layer sublimated after the sample was heated at 320 °C for only four minutes. Such a low sublimation temperature does not compare even with lowest sublimation temperatures of 400 °C observed in the aforementioned studies [38]. However, one can quickly realize that the aforementioned MoO_3_ sublimation studies were conducted under vacuum and/or in highly controlled environments [38,39], whereas our results were obtained in far less clean conditions. In other words, surface adsorbed hydrocarbons and other surface contaminants on the pristine MoS_2_ surface might have prevented the formation of a well-organized and crystalline MoO_3_ layer. Alternatively, however, the slowly grown-up MoO_3_ layer might have contained some MoO_3_ derivatives, such as molybdenum(VI) hydroxyoxides and MoO_2_(OH)_2_, which have been predicted to be formed, when water was present. If fact, the height of the formed MoO_3_ layer in Figure 5b is ca. 5 nm, which is much larger than thickness of the MoO_3_ layer obtained in air. This suggests slow formation of either an additional MoO_3_ in humid air due to Equation (2), or some additional MoO_2_(OH)_2_ species crystallizing on the sample. The MoO_2_(OH)_2_ species are far more volatile than a MoO_3_ layer [12] and sublimate at lower temperature than MoO_3_. We had no means to test for the presence of the hydroxyoxides on the MoS_2_ flake presented in Figure 5, but we decided to follow up on an unclear role of water in degradation and thermal oxidation of the MoS_2_ flakes. 

To start with, we investigated, again by AFM, the microscopic surface topography of several thermally oxidized MoS_2_ samples after their submergence in water for the times ranging from a few minutes to several hours. We did not observe any topographical changes on those previously oxidized MoS_2_ flakes. However, we could have incubated them in water for too little time in order to dissolve any MoO_3_ oxide layers and/or create any etch pits in the MoS_2_ [21]. Nevertheless, our results suggest that a thin layer of the MoO_3_ species, which originated on surfaces of the oxidized MoS_2_ flakes, prevented their further deterioration in water, at least within the time scale of several hours.

In order to start addressing the role of water during thermal oxidation of the single MoS_2_ crystals from a different perspective, we decided to perform thermal oxidation at high relative humidity. One must acknowledge that our previous thermal oxidation studies above 300 °C, even in very humid air, occurred always at local relative humidity of being almost zero per cent. This is because the saturated water vapor pressure in vicinity of a hot surface, *p_sat_(T)*, is a very strongly rising function of the temperature, so that any partial water pressure, *p*, in an open container in humid air produces a vanishingly small relative humidity, *RH* = *p* / *p_sat_(T)*, above 200 °C. Thus, in order to study an effect of relative humidity in thermal oxidation of single MoS_2_ flakes, one must utilize an enclosed chemical reactor, which assures the same pressure and relative humidity everywhere on the sample. 

**Figure 6:**
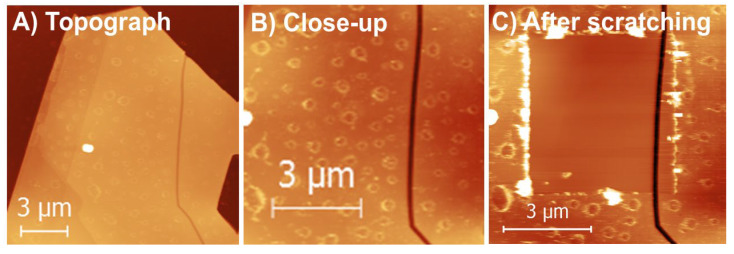
Thermal oxidation of a single MoS_2_ flake at high relative humidity. (**A**,**B**) show AFM contact mode topography of an oxidized flake and a close-up on a particular spot, which was scratched out later, in (**C**). Flake thickness depending on a spot was between 90 to 100 nm. The flake was thermally oxidized for 5 min at RH = 80 ± 7% and at a mean temperature of 205 °C. An average thickness of the scratched out layer in (**C**) was 2.2 ± 0.2 nm. Z-scales: (**A**) 274 nm; (**B**) 64 nm; (**C**) 42 nm.

In Figure 6, we present the results of thermal oxidation of single MoS_2_ crystals at *RH* = 80 ± 7%. The reaction was conducted within a custom-built chemical reactor. The sample was thermally oxidized for 5 min at mean pressure of 16 bar and mean temperature of 205 °C. It took 14 min to reach the reaction conditions and several more minutes to cool the reactor below 100 °C. A likely oxide layer was formed on the MoS_2_ samples, because we were able to scratch this layer away (see Figure 6c) at similar forces as for the data presented in Figure 3. Furthermore, the measured thickness of the likely Mo oxide layer was 2.2 ± 0.2 nm, which is also similar to the data presented in Figure 3. 

One can notice that for thermal oxidation in air, when *RH* was almost zero, the MoO_3_ oxides were not detected below 320 °C; see Figure 2. Thus, water must substantially accelerate oxidation at high relative humidity, as predicted by Equation (2). If water was acting only like a catalyst in Equation (1), then one would expect some associated triangular etch pits on the oxidized MoS_2_ surface. However, for several samples and several scratches investigated within this study we observed no triangular etch pits within the oxidized MoS_2_ layers and the underlying MoS_2_ surface, i.e., within the scratch. Thus, water does indeed induce an additional oxidation pathway, as in Equation (2). In such a case, however, a thick MoO_3_ layer would have been observed in Figure 6, which was not the case. Thus, the situation is likely more complicated.

One of the recently published studies suggested that water degrades MoS_2_ crystals by partially dissolving them to yield pits and to produce non-transparent grains of insoluble crystals of the MoO_3_ monohydrate, MoO_3_·H_2_O [21]. We have indeed observed some discoloration on the oxidized samples. Several MoO_3_ hydrates have been characterized till now [40,41,42]; in particular, MoO_3_·*n*H_2_O with *n* = 2, 1, 1/2 and 1/3. Mono and di-hydrates have been found to be most stable structurally [40,41]. The MoO_3_·2H_2_O is most stable at room temperature and MoO_3_·H_2_O was found to exist between 60 to 140 °C. Above 140 °C the MoO_3_ hydrates convert to α-MoO_3_ [41]. We have conducted our experiments above 200 °C, i.e., where any hydrates shall already convert to oxides. The only way for them to appear in our experiments would be to form exclusively at room temperature or during reactor cooling. The MoO_3_ hydrates, however, are typically produced via crystallization from slowly acidified solutions of molybdate ions [40,41,42]. Thus, in order for the MoO_3_ hydrates to appear, the molybdate ions, such as MoO_4_^2−^, need to be produced first. These anions are typically produced from reactions with MoO_3_ in alkali solutions, which is not the case in our oxidation conditions. Consequently, we are of the opinion that no MoO_3_ hydrates have been produced on MoS_2_ in humid air. 

Despite a predicted lack of the MoO_3_ hydrates, we suggest that what it takes place in high relative humidity conditions is an accelerated production of the MoO_3_ via Equation (2), as in bulk, but together with substantial conversion of the MoO_3_ layer to volatile MoO_2_(OH)_2_ species. Said hypothesis agrees with the following observations. Smolik et al. reported experimental data and thermodynamical calculations on oxidation, volatilization and re-deposition of molybdenum oxide species formed from a certain molybdenum alloy between 400 and 800 °C in flowing air [43]. The Mo oxide species on their Mo alloy underwent volatilization, which was dominated by the appearance of the MoO_3_ above 550 °C and by the appearance of the MoO_2_(OH)_2_, formed from the small ingress of water vapor, at temperatures below 550 °C. Furthermore, within conditions closer to our experimental conditions Walter et al. [12] calculated that MoO_2_(OH)_2_ is produced in thermal oxidation of the bulk MoS_2_ crystals already at 300 °C. This is much earlier than in results of Smolik et al., but similarly to Smolik et al., also for a small ingress of water vapor. Thus, it is expected that at high relative humidity appearance, and fast volatilization of the MoO_2_(OH)_2_ species occurs already at much lower temperatures than 300 °C. Consequently, at high relative humidity a crossover is expected from the MoS_2_ oxidation governed by Equation (1) to oxidation governed by Equation (2), but together with formation and sublimation of the MoO_3_ hydroxyoxides. In this way, large amounts of MoO_3_ are produced quickly and a complete basal MoS_2_ surface is transformed in Mo oxide species very fast, so that no triangular etch pits are observed. At the same time a large portion of the newly produced MoO_3_ is converted into volatile MoO_2_(OH)_2_ species, which leave the reaction environment. Overall, not much of the produced MoO_3_ stays on the MoS_2_ surface. However, systematic studies of thermal oxidation as a function of relative humidity and oxidation temperature are needed to address this hypothesis in detail. 

Finally, we compare our results with laser-induced oxidation and thinning of MoS_2_ crystals. To do so, we chose two seminal studies [44,45]. The first study discussed lased induced thinning and oxidation in air [44]. The other study was focused on laser-induced electrochemical thinning of the MoS_2_ in electrochemical, although aqueous, solutions [45]. In none of these studies, however, have Mo oxides been detected. In [44] the Authors went to great lengths to exclude any MoO_3_ presence via Raman, photoluminescence and electronic transport studies. Nevertheless, thin MoO_3_ layers are not necessarily insulating [46] and micro-Raman studies have been notorious for not showing the local presence of MoO_3_ layers [10,11]. A major reason for difficulty when trying to see any MoO_3_ on the laser-oxidized MoS_2_ flakes is certainly that laser-thinning studies are quite local. This means that only a tiny portion of each single MoS_2_ flake is heated each time and extremely small amounts of the MoO_3_ are produced. Furthermore, locally obtained temperatures achieved by laser heating of the MoS_2_ surface are likely much above sublimation temperatures of the MoO_3_ oxides. This is facilitated by poor (normal) thermal conductivity between subsequently stacked MoS_2_ layers within a crystal. This effect, however, does not play a major role for globally heated MoS_2_ samples, as in our case here. Thus, we expect different outcomes between laser-induced heating and thermally induced oxidation, and in particular more of the MoO_3_ staying on the samples oxidized thermally than in the laser-induced studies. Nevertheless, that all depends on particular experimental conditions. Consequently, it would be extremely interesting to extend the nanomechanical testing for the local presence of the MoO_3_, such as presented within our paper, to the laser-thinned MoS_2_ samples.

## 4. Conclusions

With help of XPS and AFM measurements we reported on microscopic details associated with the oxidation of thick MoS_2_ flakes deposited on silicon at temperatures between 205 and 410 °C in dry and humid air. We observed for samples oxidized at 350 °C that triangular etch pits started to coexist with MoO_3_ islands and layers. Above 370 °C the amount of MoO_3_ detected on the MoS_2_ surface started to decrease, most likely due to sublimation of the MoO_3_ layers. The MoO_3_ layers produced on the MoS_2_ samples were usually not distinguishable morphologically from an underlying MoS_2_ surface. However, the MoO_3_ layers could be scratched away from an underlying MoS_2_ surface with appropriate AFM cantilevers. We estimated breaking compressive strengths of the MoO_3_ layer to be between 0.3 and 7.8 GPa. Our results agreed with two important aspects for thermal oxidation of the MoS_2_ flakes. First, out of the several possible Mo oxides, we detected only the MoO_3_ species. Second, we suggested that MoO_3_ species had to be originally produced in the gas phase, and then only some of them adsorbed on the MoS_2_ surface. MoO_3_ adsorption has been most effective between 350 and 410 °C.

We also began to address the role of relative humidity in oxidation of the MoS_2_ crystals. We showed that sublimation of the MoO_3_ layers occurred already at 320 °C for the MoS_2_ samples oxidized previously at 370 °C and then left alone to slowly anneal at humid ambient conditions. We suggested that this result could be due to the presence of the MoO_3_ hydroxyoxides within thick MoO_3_ layers. Next, we showed the results of thermal oxidization of the MoS_2_ samples at high relative humidity of 80 ± 7% and mean temperature of only 205 °C. We observed quite dense layers of the likely Mo oxides, similarly to oxidation in air, but with no triangular etch pits within those layers and within an underlying MoS_2_ surface. We explained such results by substantially faster MoS_2_ oxidation in the presence of high relative humidity than in air and additional removal of the produced MoO_3_ oxide due to their conversion into volatile molybdenum(VI) hydroxyoxide species. 

## Supplementary Materials

The following are available online at https://www.mdpi.com/1996-1944/13/14/3067/s1, Figure S1: An example of creating ripples and scratching out an already fragmented oxide layer (due to extensive heating). Left: topography; Right: twice the uncalibrated friction, Figure S2: Progression of the process of removing already loosely bound oxides from the flake in Figure S1. Presented are 6.6 µm by 6.6 µm AFM contact mode topography images.
