# Supplementary material for: Surface-Bound and Volatile Mo Oxides Produced During Oxidation of Single MoS2 Crystals in Air and High Relative Humidity
Source: Materials (Basel). 2020 Jul 9;13(14):3067. doi: 10.3390/ma13143067 (PMC7412186; doi:10.3390/ma13143067)
Supplement: Supplementary file 1 [file materials-13-03067-s001.pdf]

materials

MDPI

Supplementary Materials: Surface-Bound and Volatile Mo Oxides Produced During Oxidation of Single MoS_2 Crystals in Air and High Relative Humidity

Robert Szoszkiewicz [1,*], Maciej Rogala [2] and Paweł Dąbrowski [2]

1 Faculty of Chemistry, Biological and Chemical Research Centre, University of Warsaw, Żwirki i Wigury 101, 02-089 Warsaw, Poland.
2 Department of Solid State Physics, Faculty of Physics and Applied Informatics, University of Lodz, Pomorska 149/153, 90-236 Lodz, Poland.

* correspondence: rszoszkiewicz@chem.uw.edu.pl

Below, we produce several more examples of scratching out a thin oxide layer during contact-mode AFM scanning at room temperaturę from previously thermally oxidized single MoS_2 flakes.

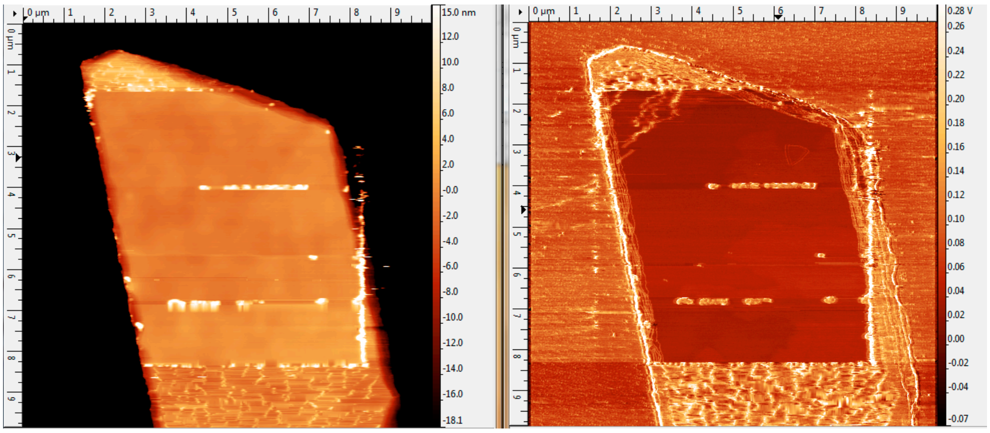

Figure S1. An example of creating ripples and scratching out an already fragmented oxide layer (due to extensive heating). Left: topography; Right: twice the uncalibrated friction.

AFM contact mode imaging, 10 µm by 10 µm. In the central zone of the MoS_2 flake heated extensively at 350 °C 6.6 µm by 6.6 µm area has been scratched out at room temperature. Figure S2 shows the progression of scratching out the oxide layer.

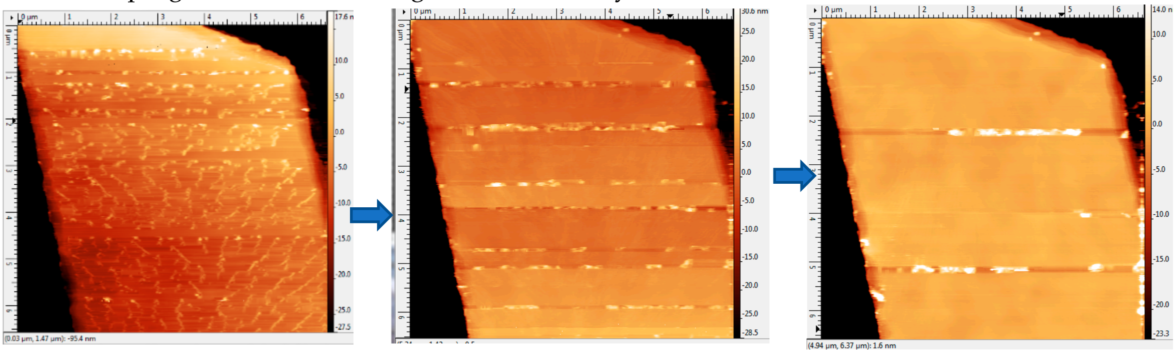

Figure S2. Progression of the process of removing already loosely bound oxides from the flake in Figure S1. Presented are 6.6 µm by 6.6 µm AFM contact mode topography images.

© 2020 by the authors. Submitted for possible open access publication under the terms and conditions of the Creative Commons Attribution (CC BY) license (http://creativecommons.org/licenses/by/4.0/).
